# Medical Society of the County of New York

**Published:** 1881-06

**Authors:** 


### MEDICAL SOCIETY OF THE COUNTY OF NEW YORK.

At a stated meeting, April 25th, Dr. Clinton Wagner read a paper on Habitual Mouth-Breathing: Its Causes, Effects, and Treatment. He first called attention to the fact that the habit of mouth breathing, with its attending evils, was confined to civilized nations, and entirely unknown among the savage races of mankind. Thus, Catlin had found that in one hundred and fifty tribes of North American Indians there was no deafness or disease of the air-passages, and after a careful study of the subject had arrived at the conclusion that this was attributable to the fact that nasal breathing was universally practiced among them. This experience was confirmed by Dr. Wagner's own more limited observations when he was an army surgeon in Idaho. Near the post where he was stationed was a large Indian village, the inhabitants of which were nominally under his professional care, and he had frequent opportunities for noticing the entire exemption of the community from ear, nose, and throat disease. Yet the sanitary conditions in which these people lived were, as a rule, of the most unfavorable character, and there could be no doubt that this immunity was due solely to the total absence of mouth-breathing among them.

The causes of this unfortunate habit, so prevalent in civilized communities, were to be looked for in the nose, mouth and throat. In regard to the conditions giving rise to it, it had formerly been impossible to arrive at a correct diagnosis in many instances ; but with the rhinoscope and other modern adpliances at our command, this was no longer the case. Among the nasal causes was mentioned occlusion of the inferior nares, particularly by a deviation of the septum, which might be either congenital or acquired. Sometimes the septum was twisted like the letter S, a condition apt to produce stenosis on both sides. Other sources of mouth-breathing were found in the presence of nasal polypi, mixomata, fibromata, adenomata, exostoses, and false membranes resulting from syphilitic or strumous ulcerations. In some individuals there was a congenital imperforate condition of the nares, and in others there was a general thickening and hypertrophy of the mucous membrane. It was not necessary, however, that the nares should be completely closed. When any one of the above pathological conditions existed, even a slight cold in the head would often render nasal breathing exceedingly difficult, and if this were to recur from time to time the pernicious habit of mouth-breathing would gradually be acquired.

Of mouth causes, chronically enlarged tonsils constituted the most common. Among the others enumerated were tumors of the soft palate and pharynx, and also elongated uvula. If the latter was present, the patient would soon find by experience that the disagreeable tickling and irritation caused by it were relieved to a great extent by opening the mouth, which always caused more or less retraction of the velum, and with it of the uvula. Irregular, uneven. and imperfect teeth also sometimes give rise to the habit of mouth-breathing.

As to the effects produced by it, they could be at once recognized in any one who had been addicted to it for any considerable time. Among the characteristics plainly stamped upon the countenance of such a one are the retracted lips, the protruding teeth, the open mouth with the peculiar wrinkles surrounding it, and the silly and idiotic expression which the observer could at once recognize. The law of compensation did not apply to the nose as to other organs. Thus, if one eye or one ear were impaired. the sensitiveness of the other became increased, while if one arm was amputated the strength and usefulness of the other was augmented ; but the closure of the meatus on one side of the nose did not cause the other one to expand. On the contrary, this one also was apt to become diminished in size. The sense of smell became greatly impaired or was altogether lost, while the whole contour of the nose was changed. The sense of hearing was more or less affected, the relaxation of the muscles controlling the mouth of the Eustachian tube, leaving this continually open ; and the trouble varied in different cases from slight to total deafness

The effects upon the pharynx were really recognized. The constant action upon the mucous membrane of cold air, which was very apt to be loaded with impurities, resulted in follicular pharyngitis or pharyngitis sicca, one of the most distressing of throat affections. The patient was forever making efforts to dislodge the hard, dry, tenacious mucous, and so common was this trouble that a continual hawking saluted our ears, whether we were in the cars or at the social gathering, the church or the theater. Chronic catarrhal laryngitis was also a common result of mouth breathing ; and when a strumous diathesis was present tubercular laryngitis was almost certain to follow. Snoring, too, was due to it, this habit being unknown among those who breath through the nose.

The effects of mouth-breathing upon the general constitution was very serious, and in young subjects it was apt to result in the malformation of the thorax known as "pigeon-breast," and in imperfect oxygenation of the blood and faulty nutrition. Again, asthma might be produced by closure of the nasal passages from any cause, and nurslings suffering from coryza are sometimes subject to asthmatic attacks during sleep. In some cases, indeed the tongue had been swallowed under these circumstances, and death had resulted from asphyxia.

Dr. Wagner then went on to speak of the treatment, and said that the local cause was first to be sought for in any case, after which such operation should be performed ro topical medication applied as was necessary to remove it. After referring to the operative procedures for correcting the shape of a deviated septum and removing exostoses, polypi, and other forms of tumor, he advocated very strongly the use of dilatation by means of metallic sounds for stenosis of the lower meati from hypertrophy of the mucous membrane over the inferior turbinated bones. In this plan of treatment, which he had found very successful, he had been guided by the same principles which had proved of service in urethal surgery. At first the passage of the instrument proved quite painful in some cases, but not more so than the introduction of the sound into the bladder often proved. This hyperæsthesia passed off after a few applications, however, and the patient generally derived so much relief from the procedure that he was quite willing to have it repeated as often as was necessary. The dilators employed varied in size from that of an ordinary pocket case probe to that of a No. 6 or 8 urethal sound; and weeks, and sometimes months, were required for the process. For the first week it was best to introduce the instrument daily, but afterwards less often. Patients could also be instructed to practice the dilatation themselves in any case where this was desirable. In addition it was often advisable to make the following application to the mucous membrane :

| | |
|---|---|
| R Iodinii . . . . . . . | gr. ij. |
| Potass. iodidi . . . . . | gr. iv. |
| Zinci iodidi . . . . | gr. x. |
| Aquæ . . . . . . . | fl. ℥i. |

In the above formula the chloride of zinc (five grains to the ounce) could sometimes be substituted with advantage for the iodide. Where the tissue was excessively hypertrophied the galvano-cautery was recommended.

If the mouth breathing was due to enlarged tonsils, excision was to be regarded as the only satisfactory remedy, and Dr. Wagner stated that he never made topical applications in such cases except under protest, when urgent objection was made to the use of the knife. The operation, if performed with the guillotine, was very simple, and, in his opinion, wholly devoid of danger. When infants and very young children breathed through the mouth instead of the nose, an examination should be made to find out what the difficulty was. Mothers and nurses, the most careful to keep every other part of the child scrupulously clean, almost universally neglected the nose; and it was recommended that the noses of infants should be cleansed by means of a small syringe and warm water, or a small camel's hair brush. A little vaseline applied at night would prevent hardening of the nasal secretion until the child was old enough to learn to snuff up water with the nostrils from the hand. If the mouth was found open during sleep the lips should be gently pressed together, and all children at an early age should be taught the vital importance of always breathing through the nose instead of the mouth.

Dr. Wagner's paper was discussed by Dr. Andrew H. Smith and others, after which Dr. A. D. Rockwell read a paper entitled Progressive Locomotor Ataxia Differentiated from Functional Conditions which simulate it.—*Boston Medical and Suraical Journal.*

